# The Application of Metal–Organic Frameworks and Their Derivatives for Supercapacitors

**DOI:** 10.3390/nano10112268

**Published:** 2020-11-16

**Authors:** Simin Huang, Xue-Rong Shi, Chunyan Sun, Zhichang Duan, Pan Ma, Shusheng Xu

**Affiliations:** 1 School of Material Engineering, Shanghai University of Engineering Science, 333 Longteng Road, Songjiang District, Shanghai 201620, China; M050119126@sues.edu.cn (S.H.); M050119115@sues.edu.cn (C.S.); M050119205@sues.edu.cn (Z.D.); mapan@sues.edu.cn (P.M.); 2 Institute of Physical Chemistry, University of Innsbruck, Innrain 80-82, A-6020 Innsbruck, Austria

**Keywords:** metal–organic frameworks, supercapacitors, MOFs derivatives

## Abstract

Supercapacitors (SCs), one of the most popular types of energy-storage devices, present lots of advantages, such as large power density and fast charge/discharge capability. Being the promising SCs electrode materials, metal–organic frameworks (MOFs) and their derivatives have gained ever-increasing attention due to their large specific surface area, controllable porous structure and rich diversity. Herein, the recent development of MOFs-based materials and their application in SCs as the electrode are reviewed and summarized. The preparation method, the morphology of the materials and the electrical performance of various MOFs and their derivatives (such as carbon, metal oxide/hydroxide and metal sulfide) are briefly discussed. Most of recent works concentrate on Ni-, Co- and Mn-MOFs and their composites/derivatives. Conclusions and our outlook for the researches are also given, which would be a valuable guideline for the rational design of MOFs materials for SCs in the near future.

## 1. Introduction

To meet the portable demand of all kinds of electronics, it is essential to develop an efficient and green energy storage technology. Among various energy storage devices, supercapacitors (SCs) have drawn much attention due to its feature of high power density and rapid charge-discharge capability [1,2,3,4,5,6].

SCs work mainly through two types of charge storage mechanisms: (i) adsorption of charge and (ii) redox reactions associated with chemical changes. Carbon materials (graphene, activated carbon, carbon nanotubes, etc.) with superior electrical conductivity and excellent chemical stability, usually work in the first mechanism as the SCs electrode materials, yielding large power density and long cycling life, but flat energy density. Transition metal oxides/hydroxide and conducting polymers normally work in the second mechanism, presenting high energy density but poor cycling stability due to the distortion of the microstructure in electrode materials resulting from the continuous redox reactions [7,8,9,10]. Hence, to develop advanced SC electrode materials, especially in the case of solving the problems of low energy density and poor cycling stability and pursuing high capacitance, is of vital significance.

In essence, the fast adsorption/desorption of electrolyte ions or rapid reversible oxidation–reduction reaction in SCs requires electrode materials to present short charge/ions transfer channel and abundant active (adsorption/desorption and redox) sites. MOFs [11,12,13] and their derived materials (carbon, metal oxides/sulfides, etc.) with inheriting MOFs pore structure, can provide adequate adsorption/reactive sites due to their large specific surface area and adjustable porous structure; in addition, their wide varieties and promising electrochemical activity make them suitable for SC electrode materials [14]. Tremendous efforts go into the research of MOFs and their derivatives’ applications in SCs in the past few years, where M-BTC (BTC = 1,3,5-benzenetricarboxylate, summarized in Appendix A Table A1), M-PTA (PTA = p-benzenedicarboxylate, also called terephthalate, i.e., 1,4-benzenedicarboxylate 1,4-BDC) and zeolitic imidazole frameworks (ZIFs) are the most investigated MOF series.

Due to the rapid development of this filed and ever-growing research interests, it is impossible to include all the relevant works in the present review. Consequently, in this paper, we mainly discuss and summarize the recent development of MOFs and MOFs derivatives as electrodes in SCs since 2020 where the earlier corresponding works have been reviewed in References [15,16,17,18]. Specifically, their active constituents (such as activated carbon, transition metals, metal oxides and conducting polymers), synthesis process and electrochemical performance are given. We also present an outlook and the development direction in the near future.

In this wok, we briefly discuss the application of MOFs as SCs electrode materials from two aspects: (i) MOFs and their composites are utilized directly as electrode materials of SCs [19] and (ii) MOFs-derived porous materials, using MOFs as the precursor/template, such as carbon materials, metal oxides/hydroxide and metal sulfides. The review is arranged as follows: Section 2 demonstrates briefly the application of pristine MOFs and their composites in SCs, Section 3 concentrates on the MOFs derivatives as the SCs electrode materials, and Section 4 summarizes our conclusions and outlook.

## 2. Pristine MOFs or MOFs Composites Directly Used for SCs

With large specific surface area, MOFs can efficiently store electrolyte ions in their porous structure; particularly, the metal cations can guarantee accommodation space for electrolyte and redox active sites. Unfortunately, their low conductivity limits their application in SCs as the direct electrode materials. To sort out the problem above, generally, two approaches are employed: (i) modification MOFs itself by modifying either metal center or organic ligands or both [20,21,22,23,24]; (ii) constructing MOFs/X composites by incorporating some conductive functional components into pristine MOFs [16,17], where X can be metal [25], metal oxides/hydroxides/sulfide [26], conducting polymers [27] and other porous carbon materials (graphene, CNT, etc.).

### 2.1. Modifying MOF Itself

Many previous studies have proved that the electrical properties of MOFs in SCs can be dramatically enhanced by doping heteroatoms/ions [28,29].

**Figure 1:**
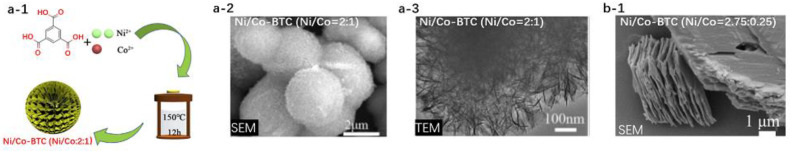
(**a**) Preparations scheme and morphology of Ni(/Co)-BTC [30] and (**b**) morphology of Ni_3−_*_x_*Co*_x_*(BTC)_2_·12H_2_O] (*x* ≈ 0.25) [31]. Reproduced with permission from Reference [30], 2020, Elsevier and [31] 2019, Elsevier.

As shown in Figure 1a and summarized in Table 1, Zhang et al. [30] synthesized spherical NiCo-BTC (Ni:Co ratio of 2:1) consisting of ultra-thin nanosheets through a one-step hydrothermal reaction. With the advantageous combination of unique structures and mixed-metallic components, the fabricated NiCo-BTC electrode shows a higher specific capacity (568 C g^−1^ at 1 A g^−1^) and better cycling stability (75.5% retention over 3000 charge/discharge cycles) than pure Ni-BTC (SC: 407 C g^−1^ at 1 A g^−1^, retention: ~35% after 3000 cycles) in 2 mol/L KOH electrolyte. The assembled asymmetric supercapacitor (ASC) with NiCo-BTC as the positive electrode and reduced graphene oxide (rGO) as the negative electrode performs a considerable energy density of 42.24 Wh kg^−1^ at the power density of 800 W kg^−1^ with eminent electrochemical cycling stability (82.6% retention of initial capacitance over 6000 charge/discharge cycles). Similar results have been found by Zhao et al. [31], earlier, who observed that bimetallic Ni_2.75_Co_0.25_(BTC)_2_·12H_2_O with loosely packed layer accordion-like structure presented the specific capacitance of 1067 F g^−1^ at a current density of 1 A g^−1^, which is superior to Ni-BTC with ~510 F g^−1^ in 3 mol/L KOH (Figure 1b).

The modification of organic ligand linkers in MOFs can be achieved by adding the second organic ligand [24] or via the post-modification of the organic ligand [32]. Zhang et al. [33] found Ni-PTA provided a specific capacitance of 721 F g^−1^ at the current density of 1 A g^−1^. However, when using the mixed ligands (PTA + BTC) as the organic linker, for example, Ni-PTA/BTC with the mole ratio of PTA:BTC of 8:2 exhibited increased specific capacitance of 920 F g^−1^ at a current density of 1 A g^−1^.

### 2.2. Constructing MOF/X Composites

#### 2.2.1. X = C

In order to tackle the poor conductivity of pristine MOFs as electrode materials, one of the most available strategies is to combine them with different types of conductive carbonaceous materials, such as graphene [34], carbon nanotubes (CNTs) [35] and carbon cloth (CC) [36], to design hybrid structure for high-performance SCs.

Previous studies [37,38] have shown that MOFs/carbonaceous-material composites yield better performance than pure MOFs. He et al. [39] stated that, compared with Ni-BPDC (BPDC = 4,4′-biphenyldicarboxylic acid), Ni-BPDC/GO (GO = graphene oxide) composite demonstrates a specific capacitance of 630 F g^−1^ at a current density of 1 A g^−1^, about 180 F g^−1^ higher than Ni-BPDC. The Ni-BPDC/GO composite yields a charge-transfer resistance (R_ct_) value of 2.23 Ω, smaller than that of Ni-BPDC with 5.96 Ω, suggesting the easier transition between ion transport and electron conduction of Ni-BPDC/GO composite. Recent works reveal that the bimetallic M-BTC and its MOF/carbonaceous-material composites, such as CuFeBTC/S-GNS (S-GNS = sulfur doped graphene nanosheet) [40] and NiCo-BTC/rGO [41], show the same trend. The NiCo-BTC/rGO with the rod-like morphology is confirmed to exhibit higher specific capacitance (958 vs. 565 F g^−1^) and longer cycle life than the NiCo-BTC electrode (109% vs. 94% of its initial capacitance over 5000 cycles at 1 A g^−1^). These results are superior to previously reported Cu-BTC (i.e., HKTST-1)/rGO which was coated on flexible carbon fiber paper (CFP) with a specific capacitance of 385 F g^−1^ at 1 A g^−1^ and pure Cu-BTC of 0.5 F g^−1^ [37]. Similar to employing polypyrrole nanotubes (PNTs) [42] or acetylene black [43] as the substrate to composite NiCo-PTA nanosheets, we recently used CC as the support to grow a dual CoNi-PTA nanosheet/nanotube [36]. The synthesized CoNi-PTA/CC (positive electrode) possessed a specific capacitance of 846 mF cm^−2^ at 1 mA cm^−2^, with a great energy density of 55.5 Wh kg^−1^ at 175.5 W kg^−1^ and remarkable cycling stability of 96.5% after 10,000 cycles.

Different from the above modification of the non-conductive MOFs, Wang et al. [44] designed conductive 3D Cu-MOF nanowire array layers by directly using 2,3,6,7,10,11-hexahydroxytriphenylene as the organic ligand linker and found the discharging time of rGO/Cu-MOF fiber electrode was almost four times longer than that of rGO fiber electrode.

#### 2.2.2. X = Metal and Metal Oxide

**Figure 2:**
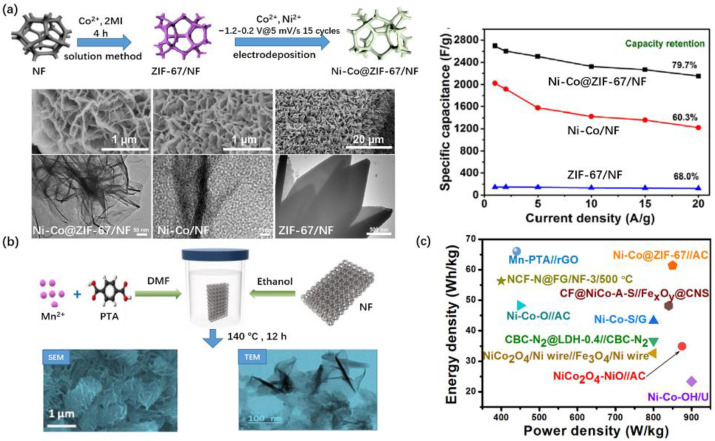
(**a**) Preparations scheme, morphology and electrical performance of Ni-Co@ZIF-67/NF arrays, Ni-Co/NF nanosheets and ZIF-67/NF nanosheets [47]; (**b**) preparations scheme and morphology of Mn-PTA/NF [48] and (**c**) their assemble SC performance. Data from References [47,48]. Reproduced with permission from References [47,48], 2020, Elsevier.

Nickel foam (NF) with a stable 3D porous structure presents high electrical conductivity, and fast mass transport capacity, ions and electrons are able to contact with the active substrate closely due to its large surface areas. Therefore, taking NF as the substrate to grow binder-free MOFs directly can greatly improve the electrode performance of pristine MOFs as the direct material for supercapacitors. Xiong et al. [45] designed a well-aligned NiO@Ni-BTC/NF to show a predominant specific capacity of 1853 C cm^−2^ at 1 mA cm^−2^, and the as-fabricated ASC device revealed a maximum energy density of 39.2 Wh kg^−1^ at 700 W kg^−1^, manifesting 94% capacitance retention over 3000 cycles. Wang et al. [46] developed a 3D triangle-like bimetallic NiCo-PTA nanosheet array on NF NiCo-PTA/NF through a facial one-step synthetic strategy by immersing a piece of nickel foam into the reaction precursor solution. Results show that the sample with Ni/Co = 3:2 delivered the advantageous specific capacity of 2230 F g^−1^ at 1 A g^−1^, exceeding most previously reported MOF-based electrode materials. The assembled NiCo-PTA/NF//AC ASC produced an energy density of 34.3 Wh kg^−1^ at a power density of 375 W kg^−1^, indicating that such a potential bimetallic NiCo-PTA nanosheets array can improve the electrochemical performances of hybrid supercapacitors. Jiang et al. [47] reports a 3D cross-porous like nano-honeycomb (Figure 2a) Ni-Co@ZIF-67/NF arrays by direct electrodeposition Ni-Co on NF supported ZIF-67 (salt: Co^2+^, organic ligand: 2 MI), which presents a specific capacitance of 2697 F g^−1^ at 1 A g^−1^, higher than Ni-Co/NF nanosheets with ~2000 F g^−1^ and ZIF-67/NF nanosheets with ~180 F g^−1^ at 1 A g^−1^, and the rate performance at 20 A g^−1^ is 79.7%. Structural analysis reveals that the Ni-Co nanosheets in Ni-Co/NF is much thicker than NiCo@ZIF-67 nanosheets in Ni-Co@ZIF-67/NF material, which, to some extent, impedes the electrons transmission. The assembled Ni-Co@ZIF-67/NF//AC ASC yields a retention rate of 80.2% after 10,000 cycles and exhibits the energy density of 61.4 Wh kg^−1^ at the power density of 853 W kg^−1^. Similarly, the ASC device assembled by the binder-free NF supported 2D layered Mn-PTA NSs (anode) with rGO (cathode) displayed even higher energy density of 66 Wh kg^−1^ at 441 W kg^−1^ [48] (Figure 2c).

Sun et al. [49] recently prepared a Cu powder decorated Mn-MOF (organic linker: 4,5-imidazole dicarboxylic acid) with a stable 3D structure via hydrothermal method. As expected, the obtained Cu/Mn-MOF yields higher specific capacitance with 1606 F g^−1^ at 0.5 A g^−1^ than Mn-MOF (1106 F g^−1^).

#### 2.2.3. X = Others

Assembling MOFs with conductive polymer can improve the electrochemical properties of the intrinsic material, as well. For instance, ZIF-67/Poly(3,4-ethylene dioxythiophene) (PEDOT) [50] delivered a higher specific capacitance of 106.8 F g^−1^ at a current density of 1 A g^−1^ than only pristine ZIF-67 electrode (34.75 F g^−1^ at 1 A g^−1^). Co-PDC (PDC: pyridine 3,5-dicarboxylate)/polyaniline (PANI) could achieve 154.9 C g^−1^ at 3 mV s^−1^ with MOF and PANI in 50/50 ratio, showing higher capacity than its pristine Co-PDC with 61 C g^−1^ [51]. The electrochemical impedance spectroscopy (EIS) measurements reveal the smaller resistance value of Co-PDC/PANI of 0.901 Ω than Co-PDC of 1.002 Ω, suggesting the higher conductivity of Co-PDC/PANI [51].

In conclusion, combining MOFs with conductive materials, such as porous carbon materials, metal oxide and polymers, has been proven to be an effective approach for designing novel supercapacitor electrode materials with outstanding performance in practical applications. The improved electrochemical properties of the composite electrode can be attributed to the synergistic effect of the composites, in which the conductive component promotes the rapid transmission of electrons, and the unique porous structure of the MOFs matrix offers a large specific surface area for adsorption and reaction. Note that the rate performance of MOFs electrode in SCs at the higher current density is not satisfying. For instance, Ni-PTA can only keep 29% at the current density ranging from 1 to 20 A g^−1^, and when using the mixed linker Ni-PTA/BTC (mole ratio of PTA:BTC of 8:2), it can raise up to 61% but still low [33]. Even when combined with GO, the specific capacitance of the Ni-BTC/rGO composite can only maintain 53.4% at the current density range from 0.66 to 3 A g^−1^ [52].

**Figure 3:**
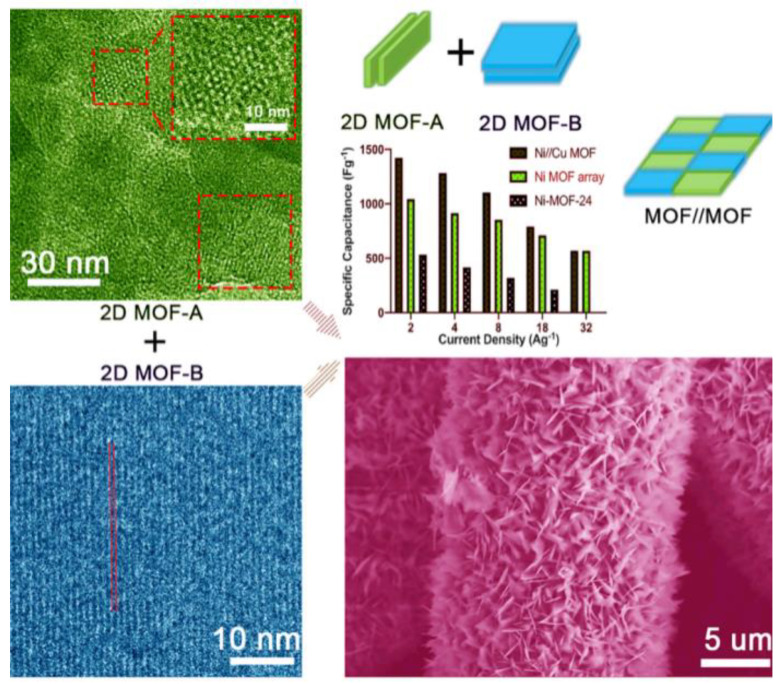
MOF//MOF strategy to prepare 2D Ni-MOF-24//Cu_3_(HITP)_2_ (Ni//Cu MOF) array. Reproduced with permission from Reference [61]. Elsevier, 2020.

It is well-known that the morphologies of materials play an essential role in their properties [53,54,55], and this is also true for MOFs’ application for SCs [56]. The morphologies of MOFs can be controlled either by surfactants, such as cetyltrimethylammonium bromide (CTAB)/polyvinylpyrrolidone (PVP), or simply by controlling the solvents. Using H_2_O as the single solvent, Ramachandran et al. [56] synthesized the mixed phased Cu@BTC-120 at 120 °C, with the octahedral shape of phase on the micro-rod structure of phase, and it showed an excellent specific capacitance of 228 F g^−1^ at 1.5 A g^−1^, superior to the previous reported single phase Cu_3_(BTC)_2_ with a specific capacitance of less than 50 F g^−1^ [37] at a current density of 4 A g^−1^ or 85 F g^−1^ at 1.6 A g^−1^, using the water/ethanol or water/DMF as solvent. Sun et al. [57] employed a solvent-controlled strategy to synthesize NiCo-MOFs (organic linker: amino-functionalized PTA) with different morphologies of nanospheres, nanosheet-assembled hollow spheres (NSHSs) and rhombus sheets. Among three morphologies, the NSHS exhibits the highest specific capacitance of 1126.7 F g^−1^ at the current density of 0.5 A g^−1^, which might be ascribed to the porous structure characteristics and large specific surface area of the NSHS. The assembled NSHS//AC ASC provides an energy density of 20.94 Wh kg^−1^ at a power density of 750.84 W kg^−1^. Du et al. [58] revealed that their prepared Co-H_6_TATAT (5,5′,5′′-(1,3,5-triazine-2,4,6-triyltriimino)tri-1,3-benzene dicarboxylic acid) material yielded a specific capacitance of 236.2 F g^−1^, and when being modified by CTAB, the value rose to 334 F g^−1^ at a current density of 1 A g^−1^. They also found Co-H_6_TATAT and Co-CTAB-6 retained 64.04% and 77.92% of the original capacitance value after 3000 cycles, respectively, demonstrating the positive effect of CTAB. Developing MOFs with 2D structures can also be regarded as a morphology-controlling technique [59,60]. Deng and co-workers [61] recently proposed a novel MOF//MOF strategy, like piecing together a puzzle, by which two kinds of 2D MOFs with specific functions are simultaneously integrated into one homogeneous layered MOF with improved electrochemical performance (Figure 3). The integrated 2D Ni-MOF-24//Cu_3_(HITP)_2_ (Ni//Cu MOF; HITP = 2,3,6,7,10,11-hexaiminotriphenylene) array delivers an excellent specific capacitance of 1424 F g^−1^, far exceeding the Ni-MOF-24 (517 F g^−1^) at the current density of 2 A g^−1^. The as-fabricated ASC can reach a maximum energy density of 57 Wh kg^−1^ and power density of 48 kW kg^−1^, respectively.

## 3. MOFs as Precursors for SCs

Metal–organic frameworks are also used as precursors or templates to prepare highly porous carbons [64,65,66,67,68,69], metal oxides/hydroxide [70,71,72], metal sulfides, metal and their composites [73], which exhibit super electrochemical performance as electrode materials for SCs.

### 3.1. MOFs Precursors for Porous Carbon

**Figure 4:**
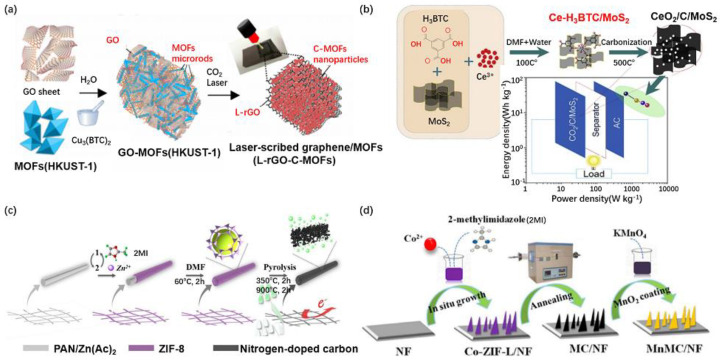
The schematic diagram of the preparation approach of (**a**) L-rGO-C-MOF [73], (**b**) CeO_2_/C/MoS_2_ (derived from Ce-BTC) [74], (**c**) ZIF-8-derived NCT [76] and (**d**) ZIF-derived MnMC on NF [77]. Reproduced with permission from References [73,74,76,77]. Elsevier, 2020.

Usually, the porous carbon materials derived from MOFs are prepared via calcination; recently, however, Van Ngo et al. [73] employed CO_2_ laser scribing to prepare novel porous 3D carbon L-rGO-C-MOF composites (a mixture of Cu_2_O, Cu and graphene revealed by X-ray diffraction XRD) by carbonizing Cu_3_(BTC)_2_ microrods and graphene (Figure 4a and Table 2). The L-rGO-C-MOF composite obtained a specific capacitance of 390 F g^−1^ at 5 mV s^−1^ and a capacity retention of 97.8% after 5000 cycles at 10 A g^−1^. The CeO_2_/C/MoS_2_ (derived from Ce-BTC) hybrid, also derived from the M-BTC subfamily, delivered an eminent specific capacitance of 1325.7 F g^−1^ and remarkable cyclic stability with capacitance retention of 92.8% over 1000 charging–discharging cycles, which is significantly higher than that of CeO_2_/C (727.5 F g^−1^) or that of MoS_2_ (300.3 F g^−1^) at 1 A g^−1^ [74] (Figure 4b). The CeO_2_/C/MoS_2_//AC (pasted on NF) ASC showed an energy density of 34.55 Wh kg^−1^ at a power density of 666.7 W kg^−1^. The better capacitive performance of CeO_2_/C/MoS_2_ hybrid is attributed to its higher surface area of 32.8 m^2^ g^−1^ than CeO_2_/C of 16.3 m^2^ g^−1^, which helps to provide more active sites for Faradaic redox reactions. Similarly, Wu et al. [75] found that combining the ZIF-8-derived hexahedral porous C with metal sulfide will yield remarkable electrochemical performance. The hexahedral porous carbon was obtained through calcination of nano-hexahedral ZIF-8 precursor at 850 °C, under argon, which was used as the support to synthesize interconnected NiS-nanosheets@porous carbon nanocomposites by a facile low-temperature water-bath method (Ni salt: tetrahydrate Ni, S source: thiourea). The as-prepared NiS@C nanocomposites exhibit the specific capacitance of 1827 F g^−1^ at 1 A g^−1^ in 2 M KOH and maintaining 72% after 5000 cycles [75]. The interconnected NiS nanosheets on the porous carbon not only provide enough available active sites to electrolyte ions but also shorten the transmission channel of ions, resulting in the competent faradaic reactions for pseudocapacitors [75].

Heteroatoms (O, N or/and P) doped carbonaceous materials derived from MOFs is also reported recently. Xu et al. [76] used polyacrylonitrile (PAN)/Zn(Ac)_2_ (Ac: acetate) fiber as the a self-sacrificing template to grow ZIF-8@PAN layer by layer, followed by removing PAN core and produce integrated ZIF-8 tubes and finally pyrolysis of ZIF-8 tubes and immersed the carbonized product in 1 M H_2_SO_4_ to obtain the nitrogen-doped carbon tubes (NCTs) (Figure 4c). The electrochemical measurement of NCTs, NCPs (N-doped carbon particles, by direct pyrolysis of ZIF-8 particles) and ACs in 1 M NaCl solution revealed the corresponding specific capacitances of ~290, 150 and 100 F g^−1^ at the current density of 1 A g^−1^. Shi et al. [78] found N, P and O co-doped Co/C composites derived from Co-hexa-(4-carboxyl-phenoxy)-cyclotriphosphazene (CTP-COOH, containing C, N, O and P) can reach a maximum of 739.6 F g^−1^ in 6 M KOH at a current density of 1 A g^−1^ and keep the capacitance retention of 80.6% over 5000 cycles. Latterly, they [79] took the same organic linker, CTP-COOH, but a different salt of Co to prepare binder-free MOFs-derived carbon as a supercapacitor electrode. They [79] claimed that the electrochemical properties of MOF-derived-C/rGO (using GO as the MOFs growth template) composites are greatly enhanced compared to bare MOF-derived C. Among the obtained Ni/C, Ni/C/rGO-x (x = 2, 4 and 8 wt%), Ni/C/rGO-4 (GO: 4 wt%) exhibits the best electrochemical performance, with a maximum capacitance of 1258.7 F g^−1^ at a current density of 8 A g^−1^ and magnificent stability with capacitance retention of 110% over 50,000 cycling tests. This is partially attributed to the increased surface area of Ni/C/GO-4 nanosheets (126.4 m^2^ g^−1^), compared to the bare Ni/C synthesized without the presence of GO with surface area of 3.7 m^2^ g^−1^.

Zhang et al. [77] also developed a binder-free MOF-derived carbon and found MnMC/NF-700 (MnO_2_ and leaf-like ZIF-67-derived nanoporous carbon on nickel foam) composites with the annealing temperature at 700 °C unveiled the specific capacitance of 531 F g^−1^ at 1 A g^−1^, with a rate capability of 85.5% in the current range of 1 A g^−1^ to 20 A g^−1^ and the capacitance retention of 82% over 5000 cycles (Figure 4d). The MnMC/NF700//AC ASC yielded an energy density of 38.78 Wh kg^−1^ at 200.01 W kg^−1^, which are substantially higher than those reported for Mn-PTA-based δ-MnO_2_//AC (23.2 Wh kg^−1^ @425 W kg^−1^) [80], MnO_x_@C@MnO_x_//NSC (NSC: N S co-doped porous 3D carbon nanocage, 23 Wh kg^−1^) and MnO_2_/CNF//Bi_2_O_3_/CNF [81,82,83]. All of these results can be ascribed to the combined impact of the 3D porous structure from nickel substrate and the outstanding electronic conductivity of ZIF-derived nanoporous carbon.

Remarkably, Javed et al. [84] found the flexible ASC assembled by pairing binder-free ultrathin Ni–Co–O NSs (NiCo_2_O_4_ revealed by XRD) and ZIF-8/ZIF-67 derived N-doped carbon NSs Ni-Co-O//NPC exhibits an energy density of 69 Wh kg^−1^ at the power density of 840 W kg^−1^ at 1 A g^−1^ in KOH hydrogel electrolyte, which is substantially higher than values reported for ASC based on the NiCo_2_O_4_ such as core–shells NiCo_2_O_4_/NiO (22.1 Wh kg^−1^ @4518.6 W kg^−1^) [85] and ZIF-derived Zn-Ni-Co hollow polyhedron (a mixture of Co_3_O_4_, ZnCo_2_O_4_, and NiCo_2_O_4_) (27.94 Wh kg^−1^ @1.3 kW kg^−1^) [86].

### 3.2. MOF Precursors for Metal Oxides/Hydroxides

Most of the recent works focus on MOFs-derived MnO_x_, CoO_x_ and NiO_x_ and their ternary metal oxide TMOs.

MnO_2_ derived from M-BTC, M-PTA and MOF-74 was recently reported. Yuan et al. [80] revealed that, among the Mn-PTA-derived α-MnO_2_, β-MnO_2_ and δ-MnO_2_, ultrathin δ-MnO_2_ with highest specific surface area of 240 m^2^ g^−1^ yielded highest specific capacitance of 416 F g^−1^ at the current density of 0.5 A g^−1^ (Table 3). Li et al. [89] utilized Ni-BTC that in situ growth on NF as a template for preparing Ni(OH)_2_-MnO_2_@C ternary composite. During the carbonization process, the graphitic carbon was generated, with the expectation of enhancing conductivity of the composite. Advantageous electrochemical performance was revealed when a self-supporting binder-free electrode was configured by the obtained Ni(OH)_2_-MnO_2_@C/NF. Specifically, about 965.1 C g^−1^ capacity was revealed at a current density of 2 mA cm^−2^, comparable to the reported gravimetric capacitance of MnO_2_-coated porous carbon fibers of 1148 F g^−1^ (areal capacitance: 3.141 F cm^−2^), where MnO_2_ was deposited on the surface of porous carbon fibers simply be immersing carbon fibers in aqueous solutions of KMnO_4_ (10 mM) at 80 °C [90]. The Ni(OH)_2_-MnO_2_@C//AC ASC showed an energy density of 39.1 Wh kg^−1^ at the power density of 221.4 W kg^−1^. The specific capacitance of Mn/Ni-MOF-74-derived spear-shaped MnNiDH was up to 2498 F g^−1^. The assembled aqueous device displays a higher energy density of 58.53 Wh kg^−1^ than 30.63 Wh kg^−1^ of the all-solid-state device [91].

**Figure 5:**
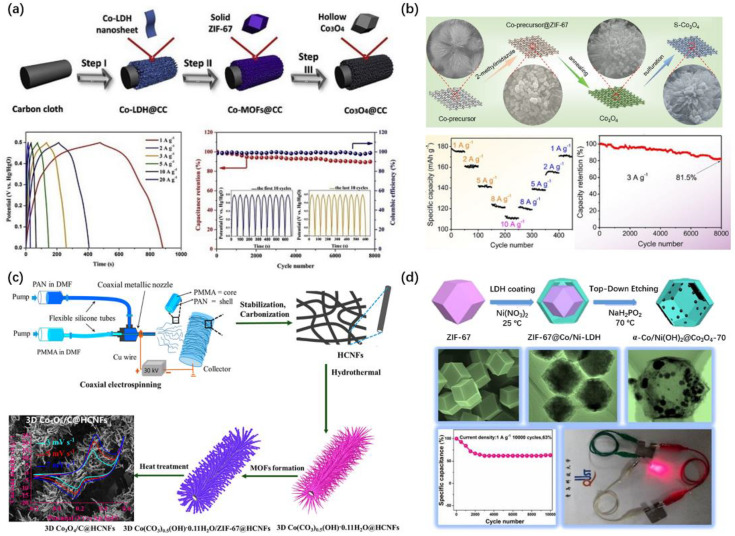
Schematic illustration for the preparation and electrochemical properties of (**a**) hollow Co_3_O_4_ arrays on carbon cloth: Step I, electrochemical deposition; Step II, in situ growth; and Step III, two steps annealing [92]. (**b**) S-Co_3_O_4_ electrode [93], (**c**) 3D Co_3_O_4_/C@HCNFs nanocomposite [94] and (**d**) α-CoNi(OH)_2_@Co_3_O_4_-70 nanocage [95]. (**a**,**b**,**d**) Elsevier, 2020. (**c**) American Chemistry Society, 2020.

CoO_x_, NiO_x_ and their ternary TMOs are mainly derived ZIF subfamily. As a binder-free electrode, binder-free 3D hollow Co_3_O_4_ polyhedral arrays [92] on CC Co_3_O_4_-60@CC derived from ZIF-67, with the in situ growth reaction time of 60 min in the step II, delivers high specific capacitance of 806 F g^−1^ at the current density of 1 A g^−1^ (Figure 5a). Another binder-free work [93] revealed that sulfur doping can increase electronic conductivity of Co_3_O_4_ on NF to decrease the electrochemical impedance and improve the rate capacity. The sulfur-doped ZIF-67 derived Co_3_O_4_ on NF (S-Co_3_O_4_/NF), prepared via the hydrothermal, annealing and sulfurization methods (Figure 5b), delivered superior specific capacity of 178 mAh g^−1^ (1424 F g^−1^) at 1 A g^−1^ and good cycling stability (81.5% capacitance retention after 8000 cycles at 3 A g^−1^). Mukhiya et al. [94] synthesized binder-free 3D porous Co_3_O_4_/C@HCNFs (HCNFs = hollow carbon nanofibers) by the preparation of graphitic-carbon-intermingled porous Co_3_O_4_ nanotentacles, which exhibit an excellent specific capacity of 1623 F g^−1^ at 1 A g^−1^ and long cyclic life, as well as good rate capability (Figure 5c). Bao et al. [95] utilized ZIF-67 as the template and NaH_2_PO_2_ as the etching agent to construct a series of Co_3_O_4_ embedded α-Co/Ni(OH)_2_ hollow nanocages (Figure 5d). The obtained α-Co/Ni(OH)_2_@Co_3_O_4_-70 with heterostructure yields a specific capacitance value of 1000 F g^−1^ at 1 A g^−1^, which is superior to the component alone (α-Co/Ni(OH)_2_: 392 F g^−1^ and Co_3_O_4_: 368 F g^−1^). The high specific capacitance of the above Co_3_O_4_ composites takes the following advantages: (i) The direct growth of electroactive materials on substrate (CC, NF, HCNFs, etc.) without any binder could reduce “dead volume”, thus decreasing the resistance; (ii) the MOF-derived highly porous Co_3_O_4_ nanotentacles/arrays furnish high surface area and rich active sites for redox reactions; and (iii) the inheriting myriad mesopores and interconnected channels allow for the easy access of ions and fast reaction kinetics.

Based on these four Co_3_O_4_ composites derived from ZIF-67, the assembled ASCs devices delivered close energy densities for Co_3_O_4_-60@CC//AC (25.3 Wh kg^−1^ at 1 A g^−1^, KOH), S-Co_3_O_4_/NF//AC (29.6 Wh kg^−1^ at 1 A g^−1^, KOH) and α-Co/Ni(OH)_2_@Co_3_O_4_-70//AC (23.88 Wh kg^−1^ at 1 A g^−1^, KOH) [95], which are smaller than the value of Co_3_O_4_/C@HCNFs//NGH (NGH: nitrogen-doped graphene hydrogel), 36.6 Wh kg^−1^ at the power density of 471 W kg^−1^.

Using ZnCoNi-ZIF as the sacrificial template and the cobalt precursor, Raphael Ejikeme et al. [86] synthesized ternary zinc–nickel–cobalt (ZNC) hollow polyhedral and the obtained ZNC (a mixture of Co_3_O_4_, ZnCo_2_O_4_ and NiCo_2_O_4_) with porous polyhedral structure consisting of shell interconnected nanoparticles delivered about 247 F g^−1^ specific capacitance at a current density of 0.1 A g^−1^. The assembled symmetric SCs exhibited 27.94 Wh kg^−1^ at a power density of 1.3 kW kg^−1^, with an outstanding cycling stability of 99% over 5000 cycles, yielding higher capacitance, superior cycling stability and lower value of resistance than the pure ZnCo_2_O_4_, due to its composition and unique porous polyhedral structure [86]. Similarly, Gong et al. [96] synthesized the metal oxide (NiO and CoO_3_) and TMOs (NiCo_2_O_4_) individually, using MOF-74 as a precursor, and found that, among them, NiCo_2_O_4_ yielded the best capacitance, as well as cycling stability, with the specific capacitance of 684 F g^−1^ and 86% retention over 3000 cycles [96]. Flower-like MnNi_2_O_4_ was designed through a two-step synthesis route, using Mn/Ni-BDC as a precursor. It delivered an outstanding specific capacitance of 2848 F g^−1^ at 1 A g^−1^ and an excellent stability of 93.25% capacitance retention over 5000 cycles at 10 A g^−1^, due to the unique ultrathin nanosheets microstructures. The assembled MnNi_2_O_4_//AC ASC provided a large energy density of 142.8 Wh kg^−1^ at a power density of 800 W kg^−1^ [98].

Similar to the preparation of pristine MOFs, the morphology and the particle size of MOF-derived MO_*x*_ used for SCs electrodes can also be controlled. An interesting pillar-coordinated strategy was recently reported that utilizes pillar ions, such as BO_2_^−^ [100] and ClO_4_^−^ [97]. Another recent interesting research topic is to create vacancies during the preparation progress. For example, a g-C_3_N_4_-coated oxygen-vacancies-rich ZnO nanocomposite (OZCN) was obtained from direct thermal decomposition of ZIF-8 (Zn-2MI) and melamine, in the air [99]. Compared with reported ZnO/g-C_3_N_4_ composites, the oxygen-vacancies-rich-ZnO/g-C_3_N_4_ (with oxygen vacancy content of ZnO up to 50.93%) electrode materials delivered a higher specific capacitance, achieving 3000 F g^−1^ at 3 A g^−1^ with an excellent cycling capability of 95.6% specific capacitance retention over 1000 cycles. The as-assembled OZCN//AC ASC resulted in a high capacitance (680 F g^−1^ at 3 A g^−1^) and a large power density and energy density (100.9 Wh kg^−1^), owing to the synergy of g-C_3_N_4_ and oxygen-vacancies-rich ZnO.

### 3.3. MOF Precursors for Metal Sulfide Composites

Transition metal bimetallic sulfides derived from MOFs show great promise for SCs applications, attracting huge attention due to their higher electrical conductivity and better electrochemical activity, when compared with their metal oxide. Liu et al. [102] developed binder-free leaf-like Co-based-ZIF-reinforced Co_9_S_8_ nanowire arrays on NF that exhibited a specific capacitance of 4.48 F cm^−2^ at 2 mA cm^−2^, superior to the capacitance values of sulfidized bare NF, and Co_3_O_4_ at the same current density, which are only about 30% and 20% of that of Co_9_S_8_, respectively, and demonstrating an exceptional cycling stability, with a capacitance loss of only 5.1 × 10^−4^% per cycle, at 25 mA cm^−2^ for 100 k cycles test.

**Figure 6:**
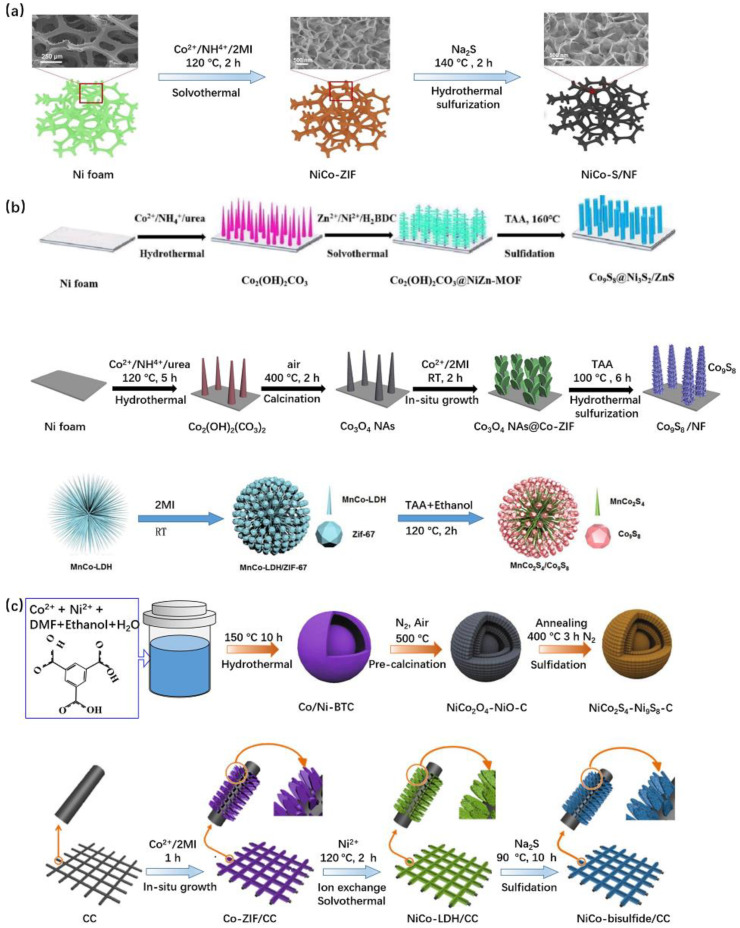
Schematic illustration of the preparation of metal sulfide (**a**) one-step synthesis of MOFs and one-step sulfidation [103], (**b**) multi-step synthesis of MOFs and one-step sulfidation b1 [108], b2: [102], b3: [106]; and (**c**) one-step synthesis of MOFs and multi-step to obtain sulfide from MOFs c1 [6], c2: [104]. Reproduced with permission from References [102,104,106]. Elsevier, 2020. Reproduced with permission from References [103,108]. Royal Society of Chemistry, 2020.

As summarized in Figure 6, to control the morphology of MOFs-derived metal sulfide, several approaches are employed to prepare transition metal sulfides: (1) one step to obtain MOFs and one step to get transition metal sulfides from MOFs (Figure 6a); (2) multi-step to synthesize MOFs, using metal oxide/LDH as the precursor, to control the morphology, and one step of sulfidation (Figure 6b); and (3) one step to grow MOFs and multi-step to acquire transition metal sulfides through metal oxide/LDH (Figure 6c). Zheng et al. [103] reported a two-step MOF-involved strategy to synthesize binder-free ultrathin nickel–cobalt sulfide nanosheet arrays on NF (NiCo-S/NF) with strong adhesion (Figure 6a). The synthesized NiCo-S/NF (with Ni:Co = 1:1, a mixture of NiCo_2_S_4,_ Co(OH)_2_ and Ni_3_S_2_) electrode demonstrated the highest specific capacitance of 3724 F g^−1^ at 1 A g^−1^ among all the discussed electrode materials in this review (Table 4). This superior capacity can be attributed to the presence of Co(OH)_2_ and the synergy between bimetals, which greatly reduced the energy barrier differences between two redox pairs (Ni^2+^/Ni^3+^ and Co^2+^/Co^3+^). The results illustrated that the in situ growth of conductive Ni_3_S_2_ on CC (NiCo-S/CC using CC as substrate, following the same preparation route with NiCo-S/NF) yields the specific capacitance of 2586 F g^−1^ at a current densities of 1 A g^−1^ [103]. However, the rate performance at the higher current density is unsatisfactory. The specific capacitance of NiCo-S/NF and NiCo-S/CC composites can only maintain ~45% and ~40% at the current density range from 1 to 20 A g^−1^. Similar binder-free NiCo-ZIF derived leaf-like NiCo-S (a mixture of Ni_3_S_2_ and Co_9_S_8_ revealed by XRD) nanosheets arrays on carbon cloth (CC) [104] NiCoS/CC electrode via NiCo-LDH/CC (Figure 6c) delivered a considerable specific capacitances of 1653 F g^−1^ at the current density of 1 A g^−1^ with 77% at current density of 20 A g^−1^, which is three time as high as the pristine NiCoS. As obtained from the EIS, the NiCoS/CC electrode had a smaller Faradaic charge-transfer resistance of 0.11 Ω than pristine NiCoS of 0.26 Ω and shorter Warburg-type line, suggesting the accelerated charge transfer and fast diffusion of electrolyte ions in the NiCoS/CC electrode. This could primarily be attributed to the sufficient diffusion paths resulting from the vertical deposition of NiCoS nanosheets on the surface of CC. Xin et al. [105] reported CoZn-ZIF-derived CoZn-S sandwiched graphene film with a high capacitance of 1640 F g^−1^ at 1 A g^−1^.

Some unique electrode structures are prepared, such as double-layer yolk–shell or polyhedrons on nanoneedles. Yan et al. [6] reported a NiCo-BTC derived double-layer yolk–shell NiCo_2_S_4_-Ni_9_S_8_-C DYMs sample showing a higher specific capacity (294 vs. 155 vs. 173 mAh g^−1^ at 1 A g^−1^) and better rate ability (81.1% vs. 78.5% vs. 52.9% from 1 A g^−1^ to 20 A g^−1^) than NiCo_2_S_4_ and Ni_9_S_8_ electrodes, respectively, and long-term cycling stability (87.3% over 5000 cycles). Jia et al. [106] recently explored a ZIF-67-derived novel hierarchical structure featuring hollow Co_9_S_8_ polyhedrons that welded on the top of a MnCo_2_S_4_ nanoneedles MnCo_2_S_4_/Co_9_S_8_ composite, which exhibited a specific capacitance of 1100.5 F g^−1^ at 1 A g^−1^ Moreover, the EIS revealed that the MnCo_2_S_4_/Co_9_S_8_ possesses a much lower charge transfer resistance of 1.9 Ω than 15.0 Ω of the MnCo_2_O_4_/Co_3_O_4_ electrode, indicating its highly conductivity, which is advantageous for the electric transport during the process of charge and discharge.

Similar to MOFs-derived metal oxide, MOFs-derived three-metal sulfides yield better electronic performance than the corresponding bimetallic sulfide. M-PTA-derived layered NiCoMoS_x_ electrode materials synthesized by Yang et al. [107] achieved a specific capacitance as high as 2595 F g^−1^ at a current density of 1 A g^−1^, which is higher than the corresponding bimetallic sulfide NiMoS_x_ with 1666 F g^−1^ and CoMoS_x_ with 1355 F g^−1^. The NiCoMoS_x_ electrode and assembled NiCoMoS_x_//AC ASC device both showed a good cycle stability, with a retention rate of 90.8% and 91.6% over 10,000 charge–discharge cycles, respectively [107]. Chen et al. [108] found that an M-PTA-derived Co_9_S_8_@Ni_3_S_2_/ZnS composite microplate array electrode showed enhanced electrochemical performance, with the areal specific capacities of 8192 and 4905 C cm^−2^ (corresponding mass specific capacity of 2427 and 1459 C g^−1^), in comparison with bare Co_9_S_8_ (2268 C cm^−2^ @ 2 mA cm^−2^; 1571 C cm^−2^ @ 5 mA cm^−2^) and layered Ni_3_S_2_ thin film (2230 F g^−1^ @ 5 mA cm^−2^ in 3 M KOH) [109].

Considering the energy density, the Co/Zn–S (CoZn-ZIF-derived CoZn–S sandwiched graphene film) @rGO//AC device reported by Xin et al. [105] shows an ultra-high energy density of 91.8 Wh kg^−1^ at the power density of 800 W kg^−1^, superior to some recently reported results, such as follow NiCo_2_S_4_/Co_9_S_8_//AC (~55 Wh kg^−1^ at 780 W kg^−1^) [110], Co_9_S_8_-Ni_3_S_2_/CC//AC (~40 Wh kg^−1^ at 379 W kg^−1^) [104], MnCo_2_S_4_/Co_9_S_8_//AC (~46 Wh kg^−1^ at 800 W kg^−1^) [106], NiCo-S/NF(NiCo_2_S_4,_ Co(OH)_2_ and Ni_3_S_2_)//AC (~45 Wh kg^−1^ at 800 W kg^−1^) [103], NiCo_2_S_4_-Ni_9_S_8_-C//rGO gel (~51 Wh kg^−1^ @1399 W kg^−1^) and NiCoMoS_x_//AC (48.2 Wh kg^−1^ @807.2 W kg^−1^).

### 3.4. MOF Precursors for Metal Phosphide Composites

**Figure 7:**
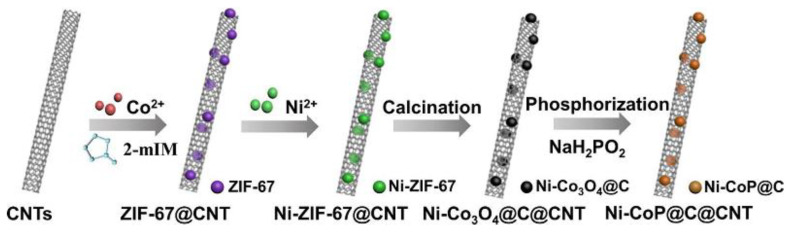
Schematic illustration of the fabrication process of Ni-CoP@C@CNT. Reproduced with permission from Reference [112]. Elsevier, 2020.

Compared with the vast investigations of the above-discussed MOFs-derived carbonaceous materials, metal oxide/hydroixde and metal sulfide, the research on MOFs-derived metal phosphide is relatively limited. The application of MOFs-derived carbon-coated CoP hollow spheres (CoP/C) [111], Ni doped CoP@C@CNT [112], N-Doped carbon-incorporated Ni_2_P/Ni [113] and yolk–shell Cu-Co-P hollow nanospheres [114] as SCs electrodes has been investigated. Taking a Ni-CoP@C@CNT nanocomposite as an example, we summarized its synthesis process and application in SCs [112]. As shown in Figure 7, Co ZIF-67 was first synthesized on CNTs, followed by Ni-doping via a solution-based ion-exchange process of ZIF-67, at room temperature; then it was calcinated to obtain Ni-doping Co_3_O_4_, and, finally, the Ni-CoP@C@CNT was developed by using NaH_2_PO_2_ as the phosphating agent. According to electronic properties calculations, Ni-doping yields an increased ratio of free electrons in Ni-CoP, resulting in better charge transmission behavior during electrochemical reactions. Consequently, the Ni-CoP@C@CNT electrode delivered a higher specific capacitance of 708.1 F g^−1^ than CoP@C of 349.2 F g^−1^ [112] but lower than the ZIF-67-derived sulfide Co_3_S_4_ of 1416 F g^−1^ at 1 A g^−1^ [110]. The Ni-CoP@C@CNT//graphene ASC advice presents an eminent electrochemical cycling stability, with a capacitance retention as high as 117% [112].

## 4. Conclusions and Outlook

This review briefly summarized and discussed the current development of MOFs, MOFs-derived porous carbon, metal oxides/composites, metal sulfides/composites and metal phosphide/composites for supercapacitors.

Several conclusions can be collected: (i) MOFs derived metal sulfide displayed an extraordinary electronic performance, especially the trimetal sulfide composites. Binder-free Ni foam (providing Ni source)-supported NiCo-S/NF (a mixture of NiCo_2_S_4_, Co(OH)_2_ and Ni_3_S_2_) derived from ZIF-67 exhibits the highest specific capacitance of 3724 F g^−1^, at a current density of 1 A g^−1^, among all the discussed materials in the work [103], lower than recently reported MnMoO_4_/NF with a super-high specific capacitance of 4609 F g^−1^ at a current density of 1 A g^−1^, synthesized from non-MOF-involved hydrothermal procedure (reactants: MnCl_2_·4H_2_O and Na_2_MoO_4_·2H_2_O) [115]; (ii) MOFs-derived metal oxide manifested a remarkable electronic performance, as well. Notably, the Ni/Mn-PTA//AC SC device (assembled by the flower-like ultrathin MnNi_2_O_4_ nanosheet derived from Ni/Mn-PTA as the positive electrode and AC as the negative electrode) yielded the largest energy density of 142.8 Wh kg^−1^ at the power density of 800 W kg^−1^, overwhelming most previously reported SCs electrode materials [98]; (iii) most of MOFs-derived porous C exhibits specific capacitance smaller than 1000 F g^−1^, while a combination with metal sulfide (MoS_2_, NiS, etc.) can enhance its behavior; for instance, NiS@C can reach a specific capacitance up to 1827 F g^−1^ [75]; (iv) Binder-free-supported composites exhibit a better performance than MOFs or MOF derivative alone, partially due to the decreased “dead volume” in the binder-free composites, resulting in the smaller resistance.

Since two materials derived from MOF-5, i.e., Zn-PTA subfamilies, were used for the electrodes of SC (Ni–Zn–Co oxide/hydroxide yields capacitance of 946 F g^−1^ at 2 mV s^−1^ [116]; nanoporous carbons exhibits specific capacitance of above 100 F g^−1^ at 5 mV s^−1^ [117]) in 2010, great progress has been made over the years. The gravimetric capacitance of MOFs derivatives increased from above 100 F g^−1^ at 5 mV s^−1^ to 3724 F g^−1^ at a current density of 1 A g^−1^. Notwithstanding these achievements, challenges still exist for the practical utilization of MOFs and MOFs-derived materials in SCs, limiting their application in our daily life: (i) For economic consideration, it is expected that MOFs are facilely synthesized in the air. Therefore, their stability in the air should also be promoted; (ii) to obtain higher capacitance and rate property for SCs, enhancing the electrical conductivity of MOFs and MOFs-derived materials is highly in urgent. For most of the present MOFs/derivatives, the rate performance is unsatisfactory; (iii) an in-depth investigation is essential to uncover the synergetic effect in composites. First-principle calculations, together with machine-learning method [118], may be needed to find out the controlling factor and favor the future rational design of MOFs/derivatives as SCs electrodes. Lots of previous works have proved that first-principle calculation method is an effective tool to investigate the synergetic effect [119,120,121]. Machine-learning methods, such as SSISSO (sure independence screening and sparsifying operator) method, developed by Ouyang et al. [122,123], have succeeded in revealing the effects of temperature and composition on materials synthesizability and stability of inorganic compounds by figuring out the best descriptor equation of Gibbs energy, to generate thousands of temperature-dependent phase diagrams [124]. Similarly, SISSO may identify the descriptor equation of a specific capacitance, using the features (band gap, pore size, surface area, density, void fraction, etc.) of known materials, and then use the descriptor equation to predict the materials capacitance directly, once their features data are available.

The investigations show that vacancies such as Ni vacancies in Ni/NiO nanoparticle derived from Ni-PTA [125], oxygen vacancies in Co_3_O_4_ or ZnO derived from the ZIF subfamily [126] showed a positive improvement in charge storage. CeO_2−x_ films with volumetric oxygen vacancies rendering Ce^3+^ concentrations as high as ~60 at% yielded the highest volumetric capacitance of 1873 F cm^−3^ among the reported works [127]. Therefore, to create the vacancies in MOFs derivatives and investigate the influence of vacancies on the electronic performance of MOFs derivatives as the SC electrode materials could be an interesting research topic.

## Figures and Tables

**Table 1:** Selected MOF-based electrodes for supercapacitor applications. Cap., capacitance (F g^−1^); CD, current density (A g^−1^); CR, capacity retention; CN, cycle number; Ele., electrode; ED, energy density (Wh kg^−1^); PD, power density (W kg^−1^); SA, surface area (m^2^ g^−1^); PS, pore size (nm). The same below.

MOFs	Electrolyte	Morphology	Cap.	CD	CR(CN)	Ele. ^1^	ED@PD	Reference
Ni-BTC	6 M KOH	rod and particles	847.3	1				[52]
Ni-BTC/rGO	6 M KOH	rod and particles	1154.4	1	90% (3000)			[52]
NiCo-BTC (Ni:Co ~ 3:2)	1 M KOH	rod-like	565	1	94% (5000)			[41]
NiCo-BTC/rGO (Ni:Co ~3:2)	1 M KOH	rod-like	958	1	109% (5000)			[41]
NiCo-BTC (Ni:Co = 2.75:0.25)	3 M KOH	PS: 2	1067	1	68.4% (2500)			[31]
NiO@Ni-BTC/NF	3 M KOH	cage-shape	1853 ^5^	1 ^7^	94% (3000) ^SC 8^	p//CNT	39.2@700	[45]
CuFe-BTC/S-GNS		SA: 568.75; PS: 9.71	1164.3	0.5	92.5% (10,000)			[40]
{SiW_10_Mn_2_}@Mn-BTC	1 M Na_2_SO_4_	SA: 16.44; PS: 3.94	211	1	96% (5000)	p//n	1.2@211.7	[25]
Ni-BTC and TPA	6 M KOH	SA: 64.8	920	1	80% (3000)	p//AC		[33]
NiCo-PTA (Ni:Co of 2:1)	2 M KOH	Flower-like SA: 0.254	1300	1	71.0% (3000)			[21]
CoNi-PTA/CC	1 M KOH	crystal: 200 nm	0.846 ^6^	1 ^7^	96.5% (10,000)	p//gCNT	55.5@175.5	[36]
NiCo-PTA@PNTs	2 M KOH	SA: 66.5; PS: 2~5	1109	0.5	79.1% (10,000)	p//AC	41.2@375	[34]
NiCo-PTA/NF (Ni:Co = 3:2)	6M KOH	SA: 22; PS: 2.2	2230	0.5	75.2% (6000)	p//AC	34.3@375	[46]
MoS_2_@Ni-PTA	3 M KOH	SA: 462; PS: 1.3–1.4	1590.2	1	87.97% (20,000)	p//AC	72.9@375	[26]
Mn 0.1 Ni-PTA/NF	6 M KOH	nanoarray SA: 0.0182	1178 C g^−1^	0.36	80.62% (5000)	p//AC	39.6@143.8	[62]
Ni-BPDC	6 M KOH	rod-like micelles	~450	1				[39]
Ni-BPDC/GO-3	6 M KOH	macro-nanostrips	630	1	95.7% (10,000)	p//rGO	16.5@250 (5) ^9^	[39]
Ni-Co@ZIF-67/NF	2 M KOH	nano honeycomb	2697	1		p//AC	61.4@853	[47]
ZIF-67/PEDOT Co-2MI	PVA/1 M H_2_SO_4_	SA: 1926	106.8	1	93% (4000)	p//n	11@200	[50]
Co-MOF/PANI ^2^	KOH	SA: 0.016; PS: 25.06	271	0.4		p//AC	23.1@1600	[51]
Mn-MOF ^3^-Cu	6 M KOH	triangular prism	1606	0.5	83.73% (10,000)			[49]
Mn-PTA/NF	2M KOH	2D NSs SA: 202	10.25 ^6^	1	81.18% (10,000)	p//rGO	66@441	[48]
Mn-Tipa and TPA	6 M KOH	polythreaded	1357.8	1	105% (2000)	p//AC	35.8@750	[23]
NiCo-NH_2_-H_2_BDC	3 M KOH	NSHS SA: 11.66	1126.7	0.5	93% (3000)	p//AC	20.9@750.8	[57]
Ni-MOF ^4^	1 M KOH	pentagonal cone	1024.4	1	49.1% (5000)	p//AC	14.6@400	[63]
Ni-MOF-24/Cu_3_(HITP)_2_/CFP	1 M KOH	2D SA: 90	1424	2	94.3% (7000)	p//AC	57@1500 (1) ^9^	[61]

^1^ Positive electrode//negative electrode. When the material in the first column is the positive (negative) electrode in assembled SCs, it is donated as p (n). ^2^ Co-pyridine 3,5-dicarboxylate acid (MOF/PANI = 50/50%); ^3^ Mn-4,5-imidazole dicarboxylic acid; ^4^ Ni-3,5-Dicarboxyl-(3′,5′-dicarboxylazophenyl)benzene acid; ^5^ C cm^−2^; ^6^ F cm^−2^; ^7^ mA cm^−2^. ^8^ The superscript of SC means that it is the properties of assembled SC device. The same below. ^9^ If the current density in the assembled SCs test is different from the value in the fifth column, it is put in parentheses.

**Table 2:** Selected MOF-derived C-based electrodes for supercapacitor applications. MOF pr., MOF precursor; Morp., morphology. The same below.

Product	MOF Pr.	Morp.	Electrolyte	Cap.	CD	CR/CN	Electrode	ED@PD	Reference
L-rGO-C-MOF	Cu-BTC	Film SA: > 600	1M NaNO_3_	390	5 mV/s	97.8% (5000)			[73]
CeO_2_/C/MoS_2_	Ce-BTC	SA: 32.8	2 M KOH	1325.7	1	92.8% (1000)	p//AC	34.6@666.7	[74]
NiS@C	ZIF-8	Porous C	2 M KOH	1827	1	72% (5000)	p//HPC	21.6@400	[75]
Co-NC	Co-BTC	SA: 206	6 M KOH	310	0.5	87% (1200)	p//AC		[87]
NCT	ZIF-8 tube	SA: 1323.5	1 M NaCl	290	1				[76]
NCP	ZIF-8 particle	SA: 735.5	1 M NaCl	150	1				[76]
NPC@CFP	ZIF-8/ZIF-67: 50/50	ultrathin NSs	PVA/KOH hydrogel	201 ^SC^	0.55 ^SC^	90% (20,000) ^SC^	NiCo_2_O_4_//n	69@840	[84]
MnMC/NF	ZIF-67	nanoflakes	1 M Na_2_SO_4_	531	1	82% (5000)	p//AC	38.8@200	[77]
Ni-C	Ni-BDC	nanofiber	6 M KOH	672	2	57% CD: 2–10	p//n	17.8@350	[88]
N, P and O co-doped Ni/C	Ni-CTP-COOH	SA: 3.7	6 M KOH	~240	8				[79]
N, P and O co-doped Ni/C/rGO	Ni-CTP-COOH/GO	SA: 126.4 NS	6 M KOH	1258.7	8	110% (5000)	p//AC	79.7@1275	[79]
N, P and O co-doped Co/C	Co-CTP-COOH	SA: 16 PS: 5	6 M KOH	739.6	1	80.6% (5000)	p//AC	30.4@800	[78]

**Table 3:** Selected MOF-derived MO_*x*_-based electrodes for SC applications.

Product	MOF Pr.	Morp.	Electrolyte	Cap.	CD	CR/CN	Ele.	ED@PD	Reference
δ-MnO_2_	Mn-PTA	SA: 240	1 M NaOH	416	0.5	60.5% (5000)	p//AC	23.2@425	[80]
MnNiDH	Mn/Ni-MOF-74	SA: 235	3 M KOH	2498	1	80.2% (10,000) ^SC^	p//AC	58.5@800 ^SC^	[91]
Co_3_O_4_	MOF-74 ^1^	SA: 48.9	1 M KOH	181.5	0.5	86% (3000)@10			[96]
Co_3_O_4_@CC (−60)	ZIF-67 (Co-2MI)	array SA: 16.23	2 M KOH	806	1	86.5% (4000) ^SC^@5 A g^−1^	p//AC	25.3@752	[92]
S-Co_3_O_4_@NF	ZIF-67 (Co-2MI)	Follower-like	KOH/PVA	1424	1	81.5% (8000)@3 A g^−1^	p//AC	29.6@804	[93]
Co_3_O_4_/C@HCNFs	ZIF-67 (Co-2MI)	nanotentacles SA: 225.7	2 M KOH	1623	1	85.2% (7000)	p//NGH	36.6@471	[94]
α-CoNi(OH)_2_@Co_3_O_4_-70	ZIF-67 (CoNi-2MI)	SA: 153.6	6 M KOH	1000	1	72.3% (8000)	p//AC	23.88@75	[95]
NiO	Ni-MOF ^2^	SA: 148.9 PS: 42.5	2 M KOH	1863	0.5	82% (5000) ^SC^	p//AC	38.4@400	[97]
NiO	MOF-74 ^3^	SA: 227.5 PS: 4	1 M KOH	105	0.5				[96]
NiCo_2_O_4_	MOF-74	SA: 59.6 PZ: 10	1 M KOH	684	0.5	86% (3000)@10 A g^−1^			[96]
MnCo_2_O_4_/Co_3_O_4_	MnCo-LDH/ZIF-67	hollow structure	6 M KOH	~838	1				[86]
MnNi_2_O_4_	Ni/Mn-PTA	NS SA: 50.80	6 M KOH	2848	1	93.25% (5000)@10 A g^−1^	p//AC	142.8@800	[98]
Co_3_O_4_, ZnCo_2_O_4_ and NiCo_2_O_4_	ZIF-67 (ZnNiCo-2MI)	Polyhedron SA: 65.9	6 M KOH	247	0.1	99% (5000)	p//n	27.9@1300	[86]
ZnO_x_/g-C_3_N_4_	TRD-ZIF-8 (Zn-2MI and CTAB)	SA: 8.59	3 M KOH aq	3000 (680)	3	95.6% (1000)	p//AC	100.9@1740	[99]
α-Ni(BO_2_^−^)-LDH	Ni-PTA	SA: 463.1 PS: 3.5–21	6 M KOH	1760	1	61.1% (10,000)	p//rGO	56.5 @111	[100]
Ni(OH)_2_-MnO_2_@C/NF	Ni-BTC	SA: 204.1	1 M KOH	965.1 C g^−1^	2 ^4^	93.90% (5000)	p//AC	39.1@221.4	[89]
NiFe_2_O_4_-NiCo-LDH@rGO	Fe-BTC Ni-2MI	hollow cube SA: 52.8	6 M KOH	750 C g^−1^	0.5	93% (3000) ^SC^@3 A g^−1^	p//AC	50@780	[101]

^1^ Ni-2,5-Dihydroxy-p-phenyldi-carboxylic acid; ^2^ Ni-1,4-bis(imidazol-1-yl)benzene (bib) Ni(ClO_4_)_2_·6H_2_O; ^3^ Ni-2,5-Dihydroxy-p-phenyldi-carboxylic acid; ^4^ mA cm^−2^.

**Table 4:** Selected MOF-derived MS_x_-based electrodes for supercapacitor applications.

Product	MOF Pr.	Morphology	Electrolyte	Cap.	CD	CR/CN	Ele.	ED@PD	Reference
Co_3_S_4_	ZIF-67 (Co-2MI)	SA: 62.8	6 M KOH	1416	1	66.1% (10,000)@5 A g^−1^			[110]
Co_3_S_4_@NiO	ZIF-67 (Co-2MI)	SA: 132.9	6 M KOH	1878	1	92.6% (10,000)@5 A g^−1^	p//AC	54.99@780	[110]
Co_9_S_8_-Ni_3_S_2_	ZIF-67 (Co-2MI)	powder	2 M KOH	~550	1	73% (3000)			[104]
Co_9_S_8_-Ni_3_S_2_/CC	ZIF-67 (Co-2MI)	NSs arrays	2 M KOH	1653	1	84% (3000)	p//AC	40@379	[104]
Co_9_S_8_/Ni_3_S_2_	ZIF-67 (Co-2MI)	Co_9_S_8_ NS wrapping around Co_9_S_8_ NWs on Ni_3_S_2_	6 M NaOH	4.48 ^1^	2 ^2^	51% (100,000)@25 ^2^	p//AC		[102]
MnCo_2_S_4_/Co_9_S_8_	MnCo-LDH/ZIF-67	SA: 34.5 PS: ~3.7	6 M KOH	1101	1	94.80% (5000)	p//AC	45.8@800	[106]
NiCo-S/NF (NiCo_2_S_4,_ Co(OH)_2_ and Ni_3_S_2_)	ZIF-67 (Co-2MI)	SA: 136	3 M KOH	3724	1	90% (3000) ^SC^	p//AC	44.76@800	[103]
NiCo_2_S_4_-Ni_9_S_8_-C	Co_0.5_Ni_0.5_-BTC	yolk-shell SA: 61.7 PS: 3.6 and 10	6 M KOH	2114	1	87.3% (5000)	p//rGO gel	51.0@1399.4	[6]
Zn_0.76_Co_0.24_S@rGO	ZIF (Co/Zn-2MI)	Sandwich	6 M KOH	1640	1	90.3% (8000)	p//AC	91.8@800	[105]
Co_9_S_8_@Ni_3_S_2_/ZnS	NiZn-MOF (NiZn-H_2_BDC)	SA: 48.3	2 M KOH	2427	2	79.7% (4000)	p//AC	0.377@1.517 ^3^	[108]
NiCoMoS_x_	Ni/Co-MOF (Ni/Co-PTA)	layered	3 M KOH	2595	1	91.6% (10,000)	p//AC	48.2@ 807.2	[107]

^1^ F cm^−2^; ^2^ mA cm^−2^; ^3^ mWh cm^−2^ @ mW cm^−2^.

## Appendix A

**Table A1:** The abbreviations and their corresponding full name used in this work.

Abbreviation	Full Name	Abbreviation	Full Name
NF	Ni foam	CC	carbon cloth
AC	activated carbon	CFP	carbon fiber paper
NS	nanosheets	HPC	hexahedral porous carbon
LDH	layered double hydroxide	PNT	polypyrrole nanotubes
CNT	carbon nanotube	NPC	nitrogen-doped carbon
NCT	nitrogen-doped carbon tubes	NCP	nitrogen-doped carbon particles
GO	graphene oxide	rGO	reduced graphene oxide
BTC	1,3,5-benzenetricarboxylate	PTA	p-benzenedicarboxylic acid
2MI	2-methylimidazole	PVA	polyvinyl alcohol
Tipa	tri(4-imidazolylphenyl)amine	BPDC	4,4′-biphenyldicarboxylic acid
PEDOT	poly(3,4-ethylene dioxythiophene)	HITP	2,3,6,7,10,11-hexaiminotriphenylene
PANI	Polyaniline	CTP-COOH	hexakis(4-carboxylphenoxy)cyclotriphosphazene
